# Comparative efficacy and safety of antidiabetic agents for post-transplant diabetes mellitus: a network meta-analysis

**DOI:** 10.3389/fmed.2025.1653147

**Published:** 2025-09-09

**Authors:** Shanbiao Hu, Gongbin Lan

**Affiliations:** ^1^Department of Kidney Transplantation, The Second Xiangya Hospital of Central South University, Changsha, China; ^2^Clinical Research Center for Organ Transplantation in Hunan Province, Changsha, China

**Keywords:** new-onset diabetes after transplantation, antidiabetic agents, network meta-analysis, SGLT2i, insulin

## Abstract

**Background:**

Post-transplant diabetes mellitus (PTDM) significantly compromises patient and graft outcomes. Although multiple antidiabetic agents are available, their comparative efficacy and safety profiles in this population remain uncertain.

**Methods:**

A systematic literature search was performed across PubMed, Web of Science, Embase, and Cochrane Library to identify clinical trials comparing antidiabetic therapies in PTDM patients. Risk of bias was assessed, and a network meta-analysis was conducted to estimate relative treatment effects. Treatment ranking probabilities, contribution plots, and funnel plots were used to evaluate hierarchy, study influence, and publication bias, respectively.

**Results:**

Twelve studies—including 10 randomized controlled trials (RCTs) and 2 cohort studies—encompassing 7,372 patients were analyzed. The network meta-analysis evaluated four outcomes: HbA1c, fasting plasma glucose (FPG), systolic blood pressure (SBP), and composite major adverse cardiovascular and kidney events (MACE and MAKE). Compared to placebo, insulin produced the greatest reductions in HbA1c (mean difference [MD] − 0.35, 95% CI − 0.90 to 0.20) and FPG (MD − 9.06 mmol/L, 95% CI − 18.66 to 0.53). DPP-4 inhibitors showed the most pronounced decrease in SBP (MD − 3.57 mmHg, 95% CI − 7.29 to 0.16). SGLT2 inhibitors (SGLT2i) demonstrated the strongest tendency to reduce MACE and MAKE events (MD − 1.95, 95% CI − 4.85 to 0.96). SUCRA plots indicated that insulin and SGLT2i ranked highest in glycemic control and safety profiles. Funnel plot analysis suggested a low risk of publication bias.

**Conclusion:**

Insulin and SGLT2i represent the most efficacious and safest options among antidiabetic treatments for PTDM, supporting their preferential consideration in post-transplant diabetes management. Further large-scale, head-to-head trials are warranted to strengthen these findings.

## Introduction

Post-transplant diabetes mellitus (PTDM) is a common and serious complication following kidney transplantation, with a reported incidence ranging from 2 to 53% (1, 2). PTDM adversely affects patient prognosis by increasing the risk of graft dysfunction, rejection, and cardiovascular disease — the latter remaining a leading cause of morbidity and mortality in this population (3). Additionally, PTDM is associated with reduced overall survival compared to non-diabetic transplant recipients (4). Given these substantial adverse outcomes, there is an urgent need for effective and safe management strategies tailored to PTDM, with the ultimate goal of preserving long-term graft function and improving patient quality of life.

According to current international consensus recommendations, PTDM refers to diabetes mellitus diagnosed after organ transplantation in individuals without a prior history of diabetes, using standard diagnostic criteria from the American Diabetes Association or World Health Organization: fasting plasma glucose ≥ 7.0 mmol/L (126 mg/dL), 2 h plasma glucose ≥ 11.1 mmol/L (200 mg/dL) during an oral glucose tolerance test, HbA1c ≥ 6.5%, or random plasma glucose ≥ 11.1 mmol/L (200 mg/dL) with symptoms of hyperglycemia. Diagnosis should be made in a stable clinical condition, at least several weeks post-transplant, and not during acute illness, high-dose corticosteroid use, or the immediate post-operative period (5). This terminology replaces the earlier term new-onset diabetes after transplantation (NODAT), which failed to account for undiagnosed pre-transplant diabetes and inconsistencies in diagnostic timing and criteria (6).

The pathogenesis of PTDM is multifactorial. It involves the diabetogenic effects of immunosuppressive agents—particularly corticosteroids and calcineurin inhibitors—post-transplant weight gain, pre-existing metabolic risk factors such as obesity and family history of diabetes, viral infections (e.g., hepatitis C, cytomegalovirus), and the stress response to major surgery (7). These factors collectively contribute to impaired insulin secretion and increased insulin resistance, resulting in post-transplant dysglycemia.

Management strategies for PTDM encompass several classes of antidiabetic agents, including insulin, sulfonylureas, sodium-glucose cotransporter-2 inhibitors (SGLT2i), and glucagon-like peptide-1 receptor agonists (GLP-1 RA). Insulin remains the cornerstone treatment, particularly in the early post-transplant period, due to its potent glucose-lowering effects. Oral agents such as sulfonylureas are also used but are associated with hypoglycemia risk (6). Novel agents, notably SGLT2i and GLP-1 RA, have demonstrated cardiovascular and renal benefits in the general diabetic population; however, their safety and efficacy in kidney transplant recipients are less well established (8). Current evidence is limited by small sample sizes, heterogeneous study designs, and short follow-up durations, making it challenging to determine the optimal therapy. Moreover, few randomized controlled trials (RCTs) have directly compared antidiabetic agents specifically in PTDM patients, contributing to uncertainty in clinical decision-making.

Network meta-analysis (NMA) offers a robust statistical framework for simultaneously comparing multiple interventions by integrating direct and indirect evidence from RCTs. The primary objective of this study is to systematically evaluate and compare the efficacy and safety of available antidiabetic therapies for PTDM. By synthesizing existing evidence through NMA, we aim to identify the most effective and safest treatment options, thereby guiding individualized therapy for kidney transplant recipients to optimize glycemic control, reduce adverse events, and improve both graft and patient outcomes.

## Methods

### Literature search strategy

A comprehensive literature search was performed across four major databases: PubMed, Web of Science, Embase, and the Cochrane Library, covering the period from database inception through April 2025. To maintain consistency in data extraction and analysis, only studies published in English were included. The search strategy combined keywords and Medical Subject Headings (MeSH) related to kidney transplantation, new-onset diabetes, and antidiabetic agents. Boolean operators “AND” and “OR” were applied to link terms, with search strings such as (“kidney transplantation” OR “renal transplant”) AND (“new-onset diabetes” OR “post-transplant diabetes”) AND (“antidiabetic agents” OR “hypoglycemic drugs” OR “glucose-lowering therapies”). This approach aimed to identify all relevant randomized controlled trials (RCT) and cohort studies assessing the efficacy and safety of antidiabetic drugs in patients with new-onset diabetes after kidney transplantation.

### Eligibility criteria

Studies were included if they met the following criteria: (1) RCTs and cohort studies evaluating the efficacy and/or safety of antidiabetic agents in patients diagnosed with PTDM; (2) studies comparing one or more antidiabetic medications, including but not limited to insulin, sulfonylureas, sodium-glucose cotransporter-2 inhibitors (SGLT2i), and glucagon-like peptide-1 receptor agonists (GLP-1 RA); and (3) trials reporting relevant clinical outcomes such as glycemic control, adverse events, graft function, or cardiovascular events. Exclusion criteria comprised non-randomized studies, observational designs without control groups, reviews, case reports, studies lacking sufficient outcome data, and those not specifically addressing PTDM populations. The patient population included adult kidney transplant recipients who developed diabetes post-transplantation. Interventions encompassed any pharmacological antidiabetic treatment, with comparators including placebo, standard care, or alternative antidiabetic agents. Primary outcomes focused on efficacy measures (e.g., HbA1c reduction) and safety parameters (e.g., incidence of hypoglycemia), while secondary outcomes included graft survival and cardiovascular events.

### Data extraction and quality assessment

Data extraction was performed independently by two reviewers using a standardized collection form to ensure consistency and minimize errors. Discrepancies were resolved through discussion; if consensus could not be reached, an independent adjudicator (not a co-author) made the final decision. Extracted data included study characteristics (author, year, design, and country), sample size, patient demographics (e.g., age, presence of PTDM or pre-existing diabetes), intervention and comparator details (drug class, dosage, treatment duration), and clinical outcomes related to efficacy (e.g., HbA1c, fasting plasma glucose, lipid profiles, body weight) and safety (e.g., adverse events, hypoglycemia).

The methodological quality and risk of bias were assessed independently by the same reviewers using the Cochrane Risk of Bias tool, covering domains such as random sequence generation, allocation concealment, blinding, completeness of outcome data, selective reporting, and other potential sources of bias.

For studies evaluating insulin therapy, we additionally recorded the timing of initiation and categorized it as either: (a) early post-transplant prophylactic or therapeutic use (<6 weeks post-transplant) aimed at *β*-cell rest and control of transient hyperglycemia, or (b) post-PTDM diagnosis use (≥6 weeks post-transplant) for management of established disease.

### Statistical analysis

A frequentist network meta-analysis model was employed to simultaneously compare the relative efficacy and safety of multiple antidiabetic agents. Heterogeneity across studies was assessed using the *I^2^* statistic. Ranking probabilities for each treatment were calculated to establish a hierarchy of efficacy and safety, and contribution plots were generated to visualize the influence of individual studies on overall estimates. Publication bias was examined using comparison-adjusted funnel plots to detect asymmetry. All statistical analyses were performed using Stata software.

## Results

**Figure 1:**
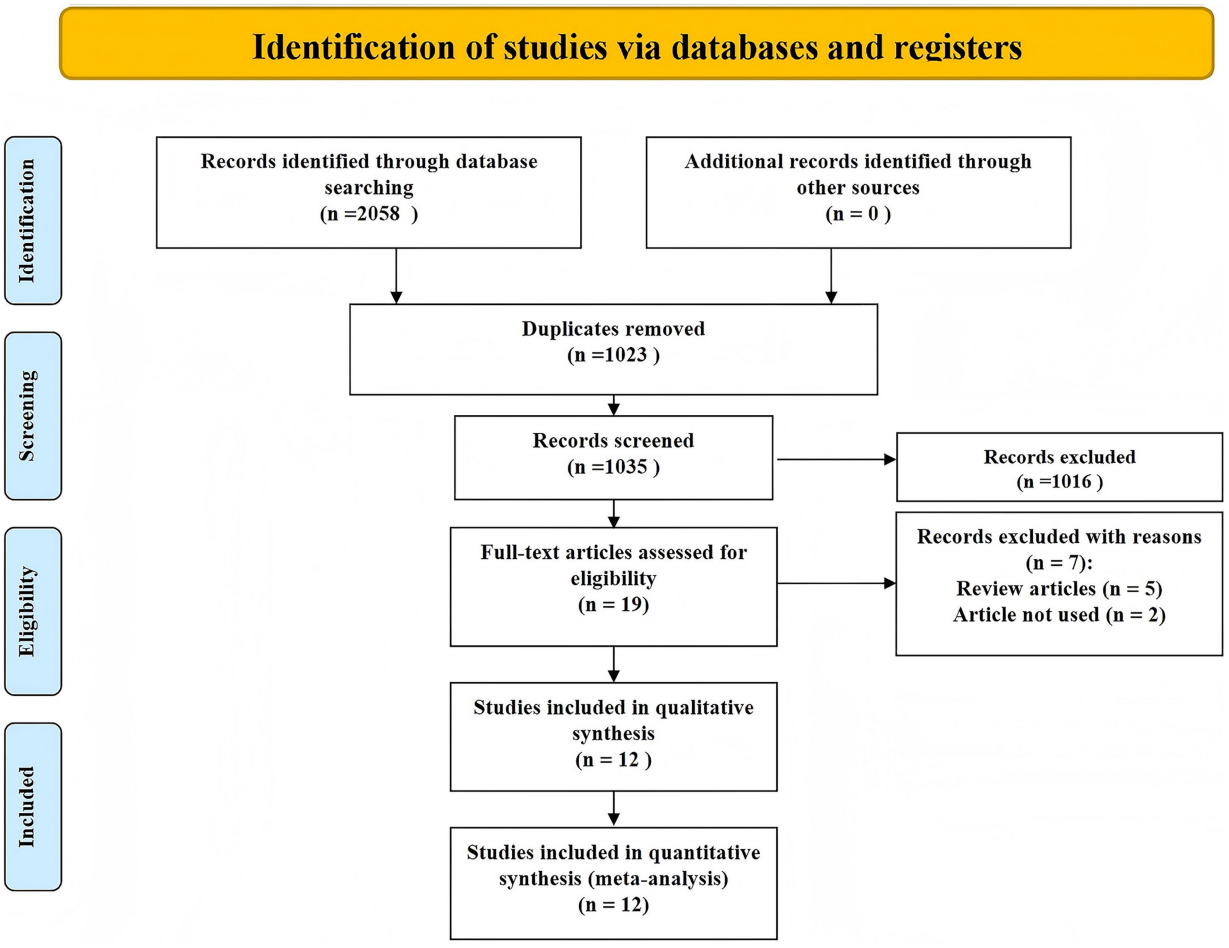
Study identification and selection.

The study selection process is detailed in the PRISMA flow diagram (Figure 1). Initially, 2,058 records were identified through database searching, with no additional records from other sources. After removing 1,023 duplicates, 1,035 unique records were screened based on titles and abstracts, leading to the exclusion of 1,016 records that did not meet the inclusion criteria. Full texts of 19 articles were assessed for eligibility, of which 7 were excluded—5 were review articles and 2 did not meet the predefined criteria for inclusion. Ultimately, 12 studies were included in both qualitative and quantitative synthesis (9–20).

Table 1 summarizes the baseline characteristics of the twelve included studies, comprising 10 RCTs and 2 cohort studies, involving a total of 7,372 patients with PTDM. These studies were conducted across multiple countries, with sample sizes ranging from 19 to 6,594 participants. Patient demographics were comparable across trials, with mean ages between 38.5 and 67 years and balanced sex distributions. Interventions evaluated included insulin, sulfonylureas, SGLT2i, GLP-1 RA, and other oral hypoglycemic agents, with treatment durations ranging from 7 days to 2.5 years. Primary outcomes consistently reported were changes in HbA1c levels, fasting plasma glucose (FPG), systolic blood pressure (SBP), major adverse cardiovascular events (MACE), and major adverse kidney events (MAKE).

**Table 1:** Characteristics of all studies and included arms.

No.	Author (year)	Study design, region	Sample of study	Participants	Age	Incretines	Treatment	Comparator	Follow-up	Outcomes
1	Johannes Werzowa (2013) (13)	RCT, Austria	48	NODAT	57.8 ± 11.8	DPP-4, TZDs	Vildagliptin: Typically 50 mg once daily; Pioglitazone: 15 mg to 45 mg once daily.	Placebo group for comparison	6 Months	HbA1c, Adverse events, 2HPG, FPG, FPI, LDL
2	Li Chun Lin (2025) (9)	Retrospective cohort study, China	6,594	DM, NODAT	57.2 ± 11.0	GLP-1 Ra	Received GLP-1 RAs during the 3 months post-transplant	Did not receive GLP-1 RAs during the 3 months post-transplant	2.5 years	Mortality, MACES, MAKE, HbA1c, Body weight, LDL, SBP, Total cholesterol, Triglycerides
3	M. Haidinger (2014) (14)	RCT, Austria	33	NODAT	64.25 ± 8.7	DPP-4 inhibitor	Vildagliptin: 50 mg once daily	Placebo group for comparison	4 Months	HbA1c, Adverse events, 2HPG, FPG, LDL, Total cholesterol, Triglycerides
4	Amin R. Soliman (2013) (15)	RCT, Egypt	45	NODAT	38.5 ± 11.5	DPP-4 inhibitor	Sitagliptin: oral sitagliptin 100 mg daily	Glargine treatment	3 Months	HbA1c, Adverse events, LDL, Body weight, Total cholesterol, Triglycerides
5	Thea Anine Strøm Halden (2019) (16)	RCT, Norway	44	PTDM	63 ± 11	SGLT2	Empagliflozin: oral 10 mg empagliflozin daily	Placebo group for comparison	6 Months	HbA1c, Adverse events, 2HPG, FPG, FPI, LDL, Total cholesterol, Triglycerides
6	Thea Anine Strøm Halden (2014) (17)	RCT, Norway	19	NODAT	67 (62–72)	DPP-4 inhibitor	Sitagliptin: dose was adjusted to renal function	Placebo group for comparison	2 Months	HbA1c (%), Adverse events, SBP, FPG
7	Balazs Odler (2023) (18)	RCT, Austria	148	PTDM	49.9 ± 13.9	Insulin	Treatment-group KTRs had 4 × daily glucose checks, received NPH insulin, and added lispro if pre-dinner glucose >140 mg/dL. Standardized protocols guided all treatments.	Usual care	24 Months	HbA1c (%), SBP, FPG
8	Srivathsan Thiruvengadam (2019) (19)	Retrospective cohort study, Australia	41	PTDM	50.3 ± 12.4	DPP-4 inhibitor	Linagliptin: oral 5 mg linagliptin daily	Usual care	2 years	FPG, HbA1c (%)
9	Elisabeth Schwaiger (2021) (20)	RCT, Austria	243	PTDM	50.7 ± 14.0	Insulin	Patients had 4 × daily glucose checks. NPH insulin was started if afternoon glucose >140 mg/dL, aiming for 110 mg/dL. Lispro was added as needed. Dosing followed a fixed protocol.	Usual care	24 Months	FPG, HbA1c (%), SBP, FPG, Adverse events
10	Johannes M. Werzowa (2018) (10)	RCT, Austria	73	NODAT	52.9 ± 13.4	Insulin	CSII with insulin lispro was initiated on day 1–2 post-transplant, with glucose-guided dose titration targeting pre-supper levels of 110 mg/dL.	Usual care	7 days	FPG, HbA1c (%)
11	Basil Alnasrallah (2019) (11)	RCT, NewZealand	19	PTDM	48.5 ± 11.6	Metformin	Metformin 500 mg twice daily	Standard care group	12 Months	FPG, HbA1c (%), Total cholesterol, Triglycerides
12	Jaehyun Bae (2015) (12)	RCT, Korea	65	NODAT	50.71 ± 12.32	DPP-4 inhibitor	DPP-4 inhibitor (vildagliptin, sitagliptin, and linagliptin)	Usual care	2 Months	FPG, HbA1c (%)

NODAT, new-onset diabetes after transplantation; PTDM, posttransplant diabetes mellitus; MACES, major adverse cardiac event; MAKE, major adverse kidney events; 2HPG, 2-Hour Postprandial Glucose; FPG, fasting plasma glucose; FPI, fasting plasma insulin; LDL, low-density lipoprotein; SBP, systolic blood pressure; HbA1c, glycated hemoglobin.

Considerable heterogeneity was observed in how PTDM/NODAT was defined, with some trials adopting American Diabetes Association (ADA) or World Health Organization (WHO) criteria, while others relied on oral glucose tolerance test (OGTT) thresholds or combined HbA1c and glucose-based criteria. The timing of diagnosis ranged from the immediate postoperative period to more than 1 year post-transplant, reflecting both early detection and late-onset cases. Insulin initiation strategies also varied markedly. In several trials (10, 18, 20), insulin was introduced within the first 6 weeks post-transplant as part of a preventive “*β*-cell rest” approach aimed at mitigating early postoperative hyperglycemia and reducing the risk of persistent PTDM. In contrast, other studies (15, 16) evaluated insulin for the management of established PTDM, typically initiated ≥6 weeks after transplantation when oral agents proved insufficient. A number of trials (9, 14) did not investigate insulin directly, instead focusing on oral hypoglycemic agents, with insulin use either excluded or reported only incidentally. The duration of insulin therapy was inconsistently reported, ranging from short-term inpatient use of 2–3 weeks to several months post-transplant, while in some studies it was not specified at all. This variability underscores the lack of standardized insulin protocols in PTDM research and highlights the influence of study design, primary endpoints, and therapeutic intent (*β*-cell rest vs. treatment of established PTDM) on clinical management strategies (Table 2).

**Table 2:** Definitions, diagnosis timing, and insulin therapy characteristics of PTDM/NODAT in included studies.

No.	Study	Disease definition	Diagnostic criteria	Post-transplant diagnosis timing	Insulin initiation timing (<6 weeks / ≥6 weeks)	Purpose of insulin therapy (β-cell rest /established PTDM)	Duration of insulin therapy
1	Johannes Werzowa (2013) (13)	Post-transplant diabetes mellitus (PTDM): New-onset diabetes following kidney transplantation, influenced by pre-existing glucose abnormalities and immunosuppressive therapy	Based on abnormal glucose metabolism screening and standard diabetes diagnostic criteria (specific glucose or HbA1c thresholds not detailed; assumed per international guidelines)	Commonly develops within the first year post-transplant; this study included T2DM diagnosed pre-transplant or within ≤3 months post-transplant	Not specified in the study	Not specified in the study (no indication whether insulin was for β-cell rest or established PTDM)	Not specified in the study (median overall follow-up was 2.5 years, but insulin therapy duration was not detailed)
2	Li Chun Lin (2025) (9)	New-onset diabetes after transplantation (NODAT), also referred to as post-transplantation diabetes mellitus, which increases cardiovascular and graft-loss risk; study participants specifically had impaired glucose tolerance (IGT) as a prediabetic condition	NODAT diagnosed per established diabetes criteria; IGT defined as 2 h plasma glucose 140–199 mg/dL on 75 g OGTT; impaired fasting glucose (IFG) defined as fasting plasma glucose 100–125 mg/dL	Patients were at least 6 months post-kidney transplantation; IGT newly diagnosed at enrollment	Not specified in the study	Not specified in the study (this trial investigated vildagliptin or pioglitazone, not insulin therapy)	Not specified in the study (3-month trial drug duration; no insulin-specific regimen reported)
3	M. Haidinger (2014) (14)	New-onset diabetes after transplantation (NODAT), a serious complication following kidney transplantation, associated with increased cardiovascular mortality and graft loss	Diagnosed by oral glucose tolerance test (OGTT) with 2 h plasma glucose ≥200 mg/dL; exclusion of prior type 1 or type 2 diabetes; stable graft function; ≥6 months post-transplant	≥6 months after kidney transplantation, newly diagnosed at enrollment based on OGTT	Not specified in the study (focus was on vildagliptin vs. placebo; no insulin regimen evaluated)	Not specified in the study (trial evaluated DPP-4 inhibitor vildagliptin, not insulin therapy; discussion mentions *β*-cell protective strategies in general)	Not specified in the study (treatment period for vildagliptin was 16 weeks; no insulin duration reported)
4	Amin R. Soliman (2013) (15)	New-onset diabetes after transplantation (NODAT), a common metabolic complication post-organ transplant, associated with increased risk of cardiovascular disease, graft failure, infection, and mortality	Defined by blood glucose ≥11.1 mmol/L after an oral glucose tolerance test; exclusion of prior type 1 or type 2 diabetes; stable graft function >6 months; BMI ≤ 40; HbA1c ≤ 8.5%	Average duration since NODAT diagnosis: 11.5 months (range 0.1–19.7 months) at enrollment	Not specifically categorized as <6 weeks or ≥6 weeks; insulin glargine was initiated in patients inadequately controlled by oral agents at randomization	For insulin glargine: glycemic control in inadequately controlled NODAT (established PTDM); no mention of *β*-cell rest	12 weeks for insulin glargine in this study
5	Thea Anine Strøm Halden (2019) (16)	Posttransplant diabetes mellitus (PTDM), a distinct type of diabetes occurring after renal transplantation, sharing traits with type 2 diabetes but influenced by immunosuppressive therapy and/or viral infections	American Diabetes Association (ADA) criteria: fasting plasma glucose ≥7.0 mmol/L, 2 h plasma glucose ≥11.1 mmol/L after 75 g OGTT, or HbA1c ≥ 6.5% (48 mmol/mol); persistent hyperglycemia ≥1 year after transplantation; no pre-transplant diabetes	≥1 year post-transplant at diagnosis (all participants had stable renal function and immunosuppressive therapy)	Not specified (<6 weeks / ≥6 weeks not reported); some patients were already on insulin prior to enrollment, with dose reductions during study	For those on insulin: management of established PTDM; no mention of β-cell rest	Not specified (empagliflozin or placebo given for 24 weeks; insulin duration prior to/during trial not detailed)
6	Thea Anine Strøm Halden (2014) (17)	New-onset diabetes after transplantation (NODAT), a common complication after kidney transplantation associated with increased cardiovascular risk and mortality	WHO criteria: fasting plasma glucose ≥7.0 mmol/L (126 mg/dL) or 2 h plasma glucose ≥11.1 mmol/L (200 mg/dL) on OGTT	>1 year post-transplant at inclusion; stable renal function; diagnosis based on database OGTT results from routine follow-up at 10 weeks and 12 months post-transplant	Not specified (<6 weeks / ≥6 weeks not reported); patients on insulin were excluded	Not applicable—insulin-treated patients excluded; study intervention was sitagliptin	Not applicable—no insulin therapy evaluated; sitagliptin administered for 4 weeks
7	Balazs Odler (2023) (18)	Post-transplant diabetes mellitus (PTDM), defined as diabetes developing after kidney transplantation in previously non-diabetic recipients	ADA criteria: 2 h OGTT plasma glucose ≥200 mg/dL (11.1 mmol/L) or HbA1c ≥ 6.5% (48 mmol/mol)	Immediately post-transplant; early postoperative period studied; follow-up up to 24 months	Initiated early postoperatively (<6 weeks) in the intervention arm of the ITP-NODAT trial	Preventive strategy for high-risk patients to reduce PTDM incidence (β-cell rest concept)	Early postoperative course—insulin given in initial months post-transplant, with follow-up for 24 months; hypoglycemia events mainly in first 3 months
8	Srivathsan Thiruvengadam (2019) (19)	Post-transplant diabetes mellitus (PTDM): hyperglycaemia developing after kidney transplantation in previously non-diabetic recipients	Fasting blood glucose >7 mmol/L or random blood glucose >11.1 mmol/L, confirmed on more than two occasions at least 48 h post-transplantation	Median 93 days post-transplant for historical cohort; median 95 days for protocol cohort (early detection emphasized)	Not specified (<6 weeks / ≥6 weeks not reported); early oral therapy with linagliptin was primary intervention; insulin used only in historical cohort when hyperglycaemia persisted	In historical cohort, insulin for management of established PTDM; protocol cohort aimed at β-cell preservation via early DPP-4 inhibitor therapy	Not specified; insulin use not protocolized, oral linagliptin therapy median initiation ~90 days post-transplant
9	Elisabeth Schwaiger (2021) (20)	Post-transplantation diabetes mellitus (PTDM), a unique form of diabetes occurring after kidney transplantation in previously non-diabetic recipients	ADA criteria at trial start; later per updated consensus guidelines: OGTT 2 h plasma glucose ≥200 mg/dL; HbA1c ≥ 6.5% used only if OGTT missed	Immediate postoperative period; early inpatient monitoring; primary endpoint assessed at 12 months, follow-up to 24 months	Initiated early postoperatively (<6 weeks) for afternoon glucose ≥140 mg/dL in the intervention arm; median initiation during hospitalization	Preventive strategy aimed at reducing PTDM incidence by controlling early postoperative hyperglycaemia (β-cell rest concept)	Duration varied per patient; insulin weaned per protocol when glucose normalized; follow-up to 24 months; majority of hypoglycaemia events occurred in first 3 months
10	Johannes M. Werzowa (2018) (10)	New-onset diabetes after transplantation (NODAT): diabetes developing in previously non-diabetic kidney transplant recipients, typically within the first year post-transplant	No history of diabetes prior to transplantation; insulin therapy initiated for pre-supper BG ≥ 140 mg/dL in early postoperative period	Immediate postoperative phase (day 1–3 post-transplant)	Initiated within <6 weeks post-transplant (typically day 1–3) when pre-supper BG ≥ 140 mg/dL	Preventive strategy against PTDM by controlling early postoperative hyperglycemia (β-cell rest concept)	Mean 19.5 days (range 2–81 days); two patients continued beyond discharge for 81 and 53 days
11	Basil Alnasrallah (2019) (11)	Post-transplant diabetes mellitus (PTDM): diabetes developing after kidney transplantation, replacing the term new-onset diabetes after transplantation (NODAT)	OGTT performed 4–12 weeks post-transplant; IGT defined as 2 h plasma glucose 7.8–11.1 mmol/L; PTDM defined per consensus guidelines and ADA criteria (OGTT ≥11.1 mmol/L, HbA1c ≥ 6.5%)	Early post-transplant (4–12 weeks) for screening; patients with IGT enrolled and followed for 12 months	Not specified (<6 weeks / ≥6 weeks not reported); no insulin therapy in study protocol	Not applicable—study intervention was metformin for IGT, not insulin; no β-cell rest or established PTDM insulin therapy involved	Not applicable—no insulin therapy; metformin given for 12 months
12	Jaehyun Bae (2015) (12)	New-onset diabetes after transplantation (NODAT), referring to diabetes developing in kidney transplant recipients with no history of diabetes prior to transplantation	Fasting plasma glucose ≥7.0 mmol/L, HbA1c ≥ 6.5%, and/or use of antidiabetic medication at 1 year after transplantation	≥1 year post-transplant for diagnosis in this study; all included patients survived >12 months after KT	Not specified (<6 weeks / ≥6 weeks not reported); only 3.1–3.4% of patients in each DPP-4 inhibitor group were on insulin	Not specified; insulin use not a focus—study evaluated DPP-4 inhibitors’ effects on glucose control and cyclosporine levels	Not specified; insulin users were few and duration was not discussed

The risk of bias assessment indicated that most included studies had a low risk in key domains such as random sequence generation and allocation concealment. However, a few studies showed unclear risk in blinding of participants and personnel due to open-label designs. Overall, attrition and reporting biases were minimal across the trials. These findings suggest that the quality of the included randomized controlled trials was generally acceptable for the purposes of this network meta-analysis (Supplementary material).

**Figure 2:**
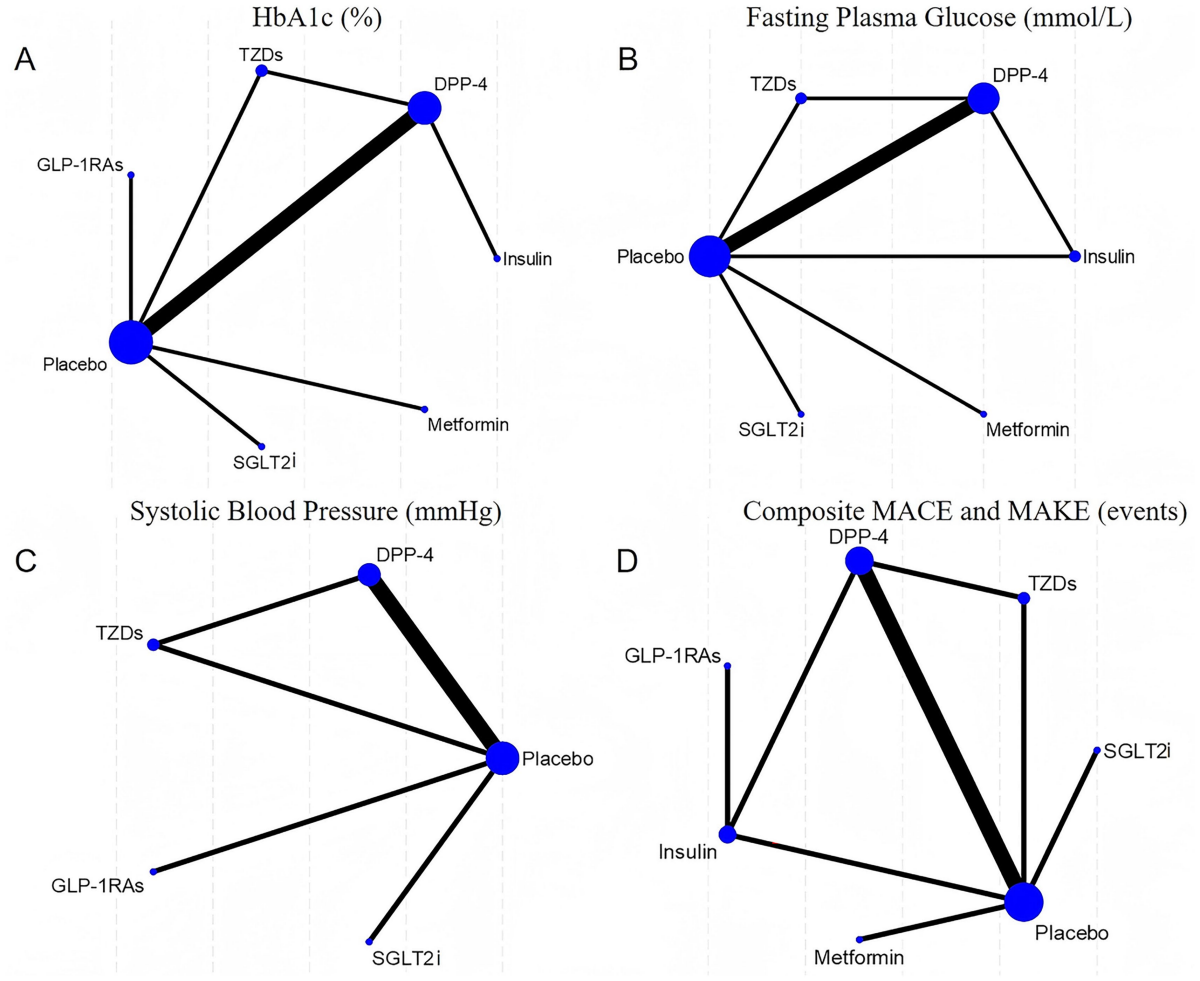
Network. **(A)** HbA1c (%); **(B)** Fasting plasma glucose (mmol/L); **(C)** Systolic blood pressure (mmHg); **(D)** Composite MACE and MAKE (events).

**Figure 3:**
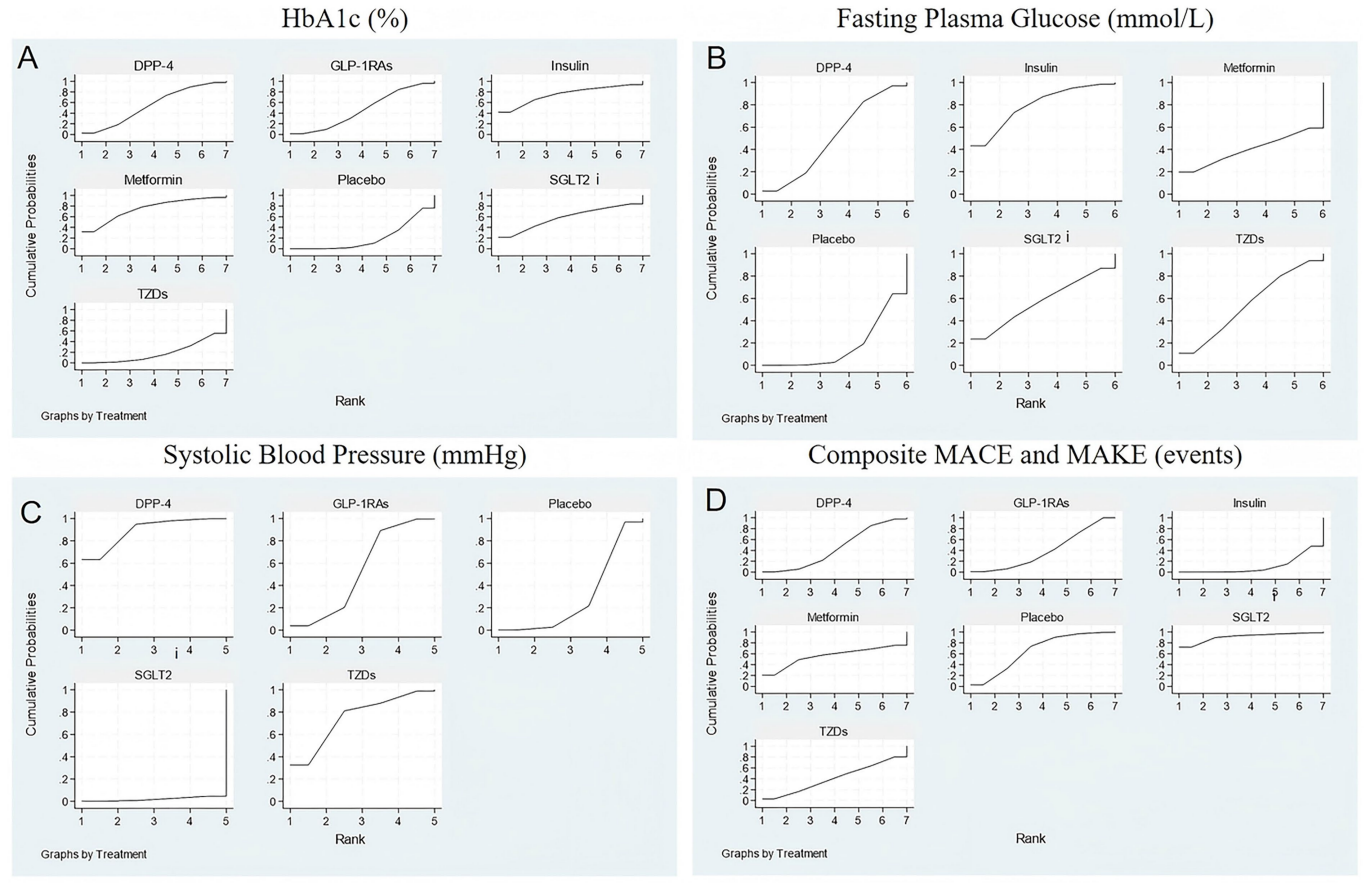
SUCRA plot for **(A)** HbA1c (%); **(B)** Fasting plasma glucose (mmol/L); **(C)** Systolic blood pressure (mmHg); **(D)** Composite MACE and MAKE (events).

Network diagrams depicting direct and indirect comparisons among the antidiabetic agents for the four evaluated outcomes are shown in Figure 2. Each network highlights the number of studies comparing treatments, indicated by node size and edge thickness. The SUCRA rankograms in Figure 3 illustrate the cumulative probabilities for treatment rankings in terms of efficacy and safety across these outcomes.

**Figure 4:**
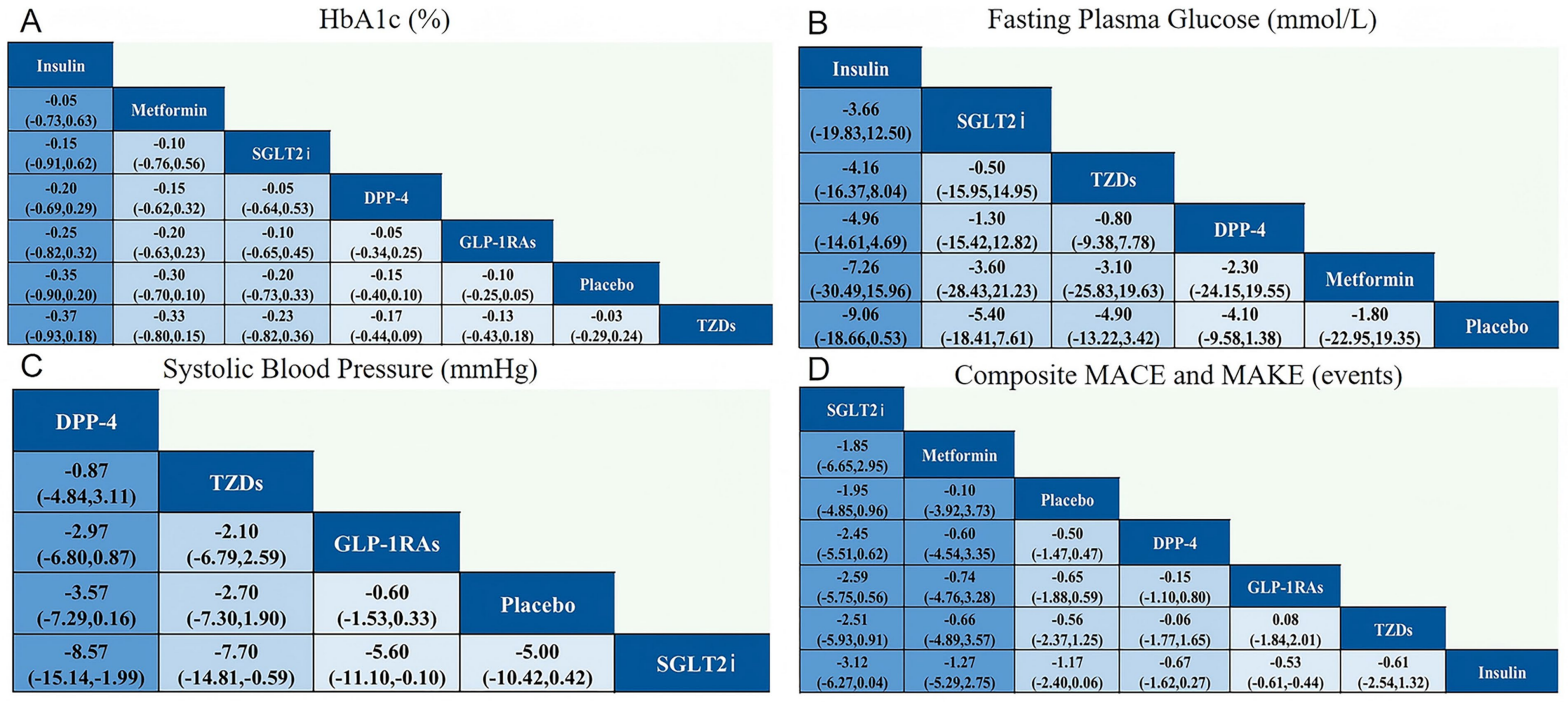
Network league for outcomes for **(A)** HbA1c (%); **(B)** Fasting plasma glucose (mmol/L); **(C)** Systolic blood pressure (mmHg); **(D)** Composite MACE and MAKE (events).

**Figure 5:**
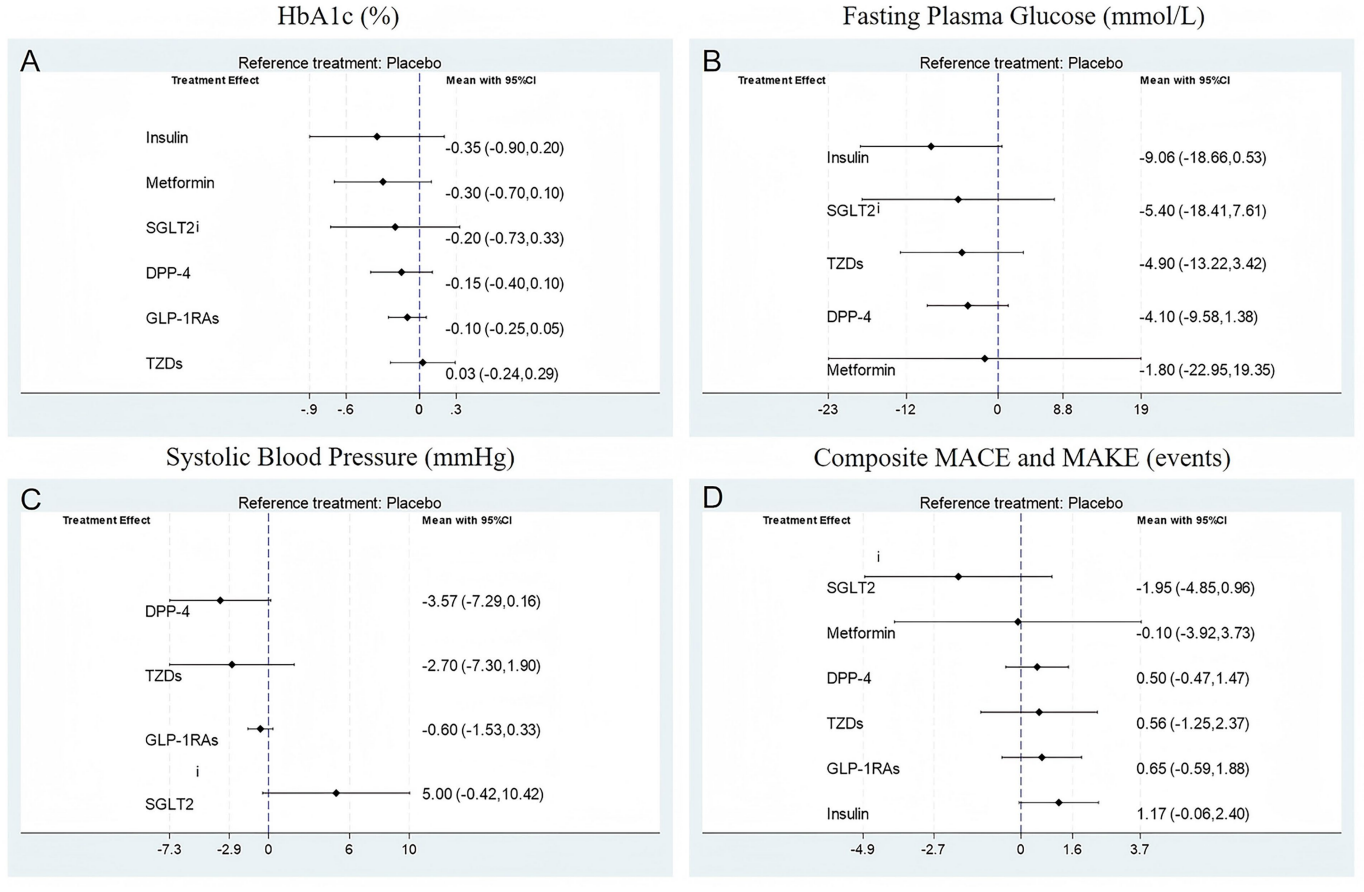
Forest plot for **(A)** HbA1c (%); **(B)** Fasting plasma glucose (mmol/L); **(C)** Systolic blood pressure (mmHg); **(D)** Composite MACE and MAKE (events).

Figure 4 displays the network league tables of comparative treatment effects among antidiabetic agents across four key outcomes: (A) HbA1c (%), (B) fasting plasma glucose (FPG, mmol/L), (C) systolic blood pressure (SBP, mmHg), and (D) composite major adverse cardiovascular and kidney events (MACE and MAKE). Corresponding forest plots are presented in Figure 5, using placebo as the reference treatment. For glycemic control (HbA1c, Figures 4A, 5A), insulin shows the most consistent reduction compared to placebo (mean difference −0.35, 95% CI -0.90 to 0.20). Other agents such as metformin, SGLT2i, DPP-4 inhibitors, and GLP-1 RAs exhibit similar small reductions in HbA1c, none reaching clear significance. This suggests insulin may have the greatest effect size on HbA1c reduction among the evaluated agents. Regarding FPG (Figures 4B, 5B), insulin again demonstrates the largest mean reduction relative to placebo (−9.06 mmol/L, 95% CI -18.66 to 0.53). Other treatments, including SGLT2i and TZDs, show smaller and nonsignificant effects. In terms of SBP (Figures 4C, 5C), DPP-4 inhibitors produced the largest reduction compared with placebo (mean difference −3.57 mmHg, 95% CI − 7.29 to 0.16), a result that approached but did not reach statistical significance. Although the absolute reduction was modest, it exceeded the typical change observed in non-transplant type 2 diabetes populations (<1 mmHg). This comparatively greater effect may be attributable to the distinct hemodynamic and pharmacologic environment in kidney transplant recipients. Nevertheless, the relatively small sample size limits the certainty of this finding, and it should be interpreted with caution. Other agents, including SGLT2i and GLP-1 RAs, show smaller and less consistent effects on SBP. For MACE and MAKE (Figures 4D, 5D), SGLT2i display the greatest reduction tendency compared to placebo (mean difference −1.95, 95% CI -4.85 to 0.96). GLP-1 RAs and other agents show smaller and nonsignificant effects. Overall, insulin appears to exert the strongest glycemic control effect, DPP-4 inhibitors show the most evident reduction in systolic blood pressure, and SGLT2i demonstrate the greatest potential for reducing adverse cardiovascular and renal events.

Assessment of publication bias through funnel plot inspection (Supplementary material) indicated symmetrical distribution of study effects around the pooled estimate, suggesting low risk of publication bias. The contribution plot (Supplementary material) revealed that a limited number of large RCTs disproportionately influenced the pooled effect estimates, underscoring their critical role in shaping the overall conclusions.

Of the insulin studies included, two trials (10, 20) initiated insulin within the first 1–2 days post-transplant as part of a beta-cell rest strategy. One study (18) initiated insulin in patients with persistent hyperglycemia beyond the early postoperative period. This heterogeneity in timing underscores that our pooled analysis reflects both early and later insulin use, rather than exclusively therapy for established PTDM.

## Discussion

This study systematically evaluated and compared the efficacy and safety of various antidiabetic agents in patients with PTDM through a network meta-analysis of 12 studies involving 7,372 patients. The analysis included treatments such as insulin, SGLT2i, DPP-4 inhibitors, and GLP-1 receptor agonists, assessing outcomes related to glycemic control, fasting plasma glucose, systolic blood pressure, and major adverse cardiovascular and kidney events. Findings indicated that insulin produced the most significant reductions in HbA1c and fasting plasma glucose, DPP-4 inhibitors showed the greatest improvement in systolic blood pressure, and SGLT2i demonstrated the strongest potential to reduce cardiovascular and renal adverse events. SUCRA rankings further supported the superior efficacy and safety profiles of insulin and SGLT2i. Overall, these results provide important evidence guiding clinical management of PTDM, suggesting insulin and SGLT2i as the most effective and safe treatment options in this population.

Compared to previous studies on antidiabetic treatments in both PTDM patients and the broader type 2 diabetes population, our findings largely align with established evidence regarding the efficacy of insulin, SGLT2i, and DPP-4 inhibitors. Prior research in type 2 diabetes has consistently demonstrated the potent glycemic control offered by insulin and the cardiovascular and renal benefits associated with SGLT2i, which is reflected in our results specific to the PTDM population (21, 22). However, some differences exist, potentially attributable to the unique characteristics of kidney transplant recipients. Factors such as immunosuppressive therapy, altered metabolism, and increased susceptibility to drug interactions in this group may influence drug efficacy and safety profiles differently than in the general diabetic population. Additionally, the pathophysiology of PTDM may differ from typical type 2 diabetes, with transplant-related stress and immunosuppressants playing a significant role in disease onset and progression. These patient-specific factors likely contribute to the observed variations and highlight the necessity of tailored therapeutic approaches for PTDM management.

Insulin exerts its glucose-lowering effect primarily by facilitating cellular uptake of glucose and suppressing hepatic glucose production, thereby directly addressing hyperglycemia common in PTDM (23). Given the insulin resistance and impaired insulin secretion observed in PTDM patients—often exacerbated by immunosuppressive agents—exogenous insulin remains a cornerstone for effective glycemic management in this population (24). SGLT2i reduce blood glucose levels by promoting renal glucose excretion through inhibition of sodium-glucose cotransporter 2 in the proximal tubules (25). Beyond glycemic control, these agents confer cardiovascular and renal benefits by mechanisms including natriuresis, reduction of intraglomerular pressure, and modulation of inflammatory and fibrotic pathways, which are particularly relevant for kidney transplant recipients at high risk of cardiovascular and renal complications. DPP-4 inhibitors enhance endogenous incretin hormone activity, increasing insulin secretion and decreasing glucagon release in a glucose-dependent manner, with a favorable safety profile and modest effects on blood pressure (26). The heart and kidney protective effects seen with SGLT2i likely arise from a combination of hemodynamic changes, improved metabolic parameters, and attenuation of oxidative stress and inflammation, making them especially valuable in the context of PTDM where cardiovascular and renal risks are amplified (27).

The clinical implications of our findings highlight the need for individualized antidiabetic strategies in PTDM, with careful consideration of both efficacy and safety. The role of insulin is strongly time-dependent. In the immediate post-transplant period (typically within the first 6 weeks), insulin is often the preferred therapy to rapidly control hyperglycemia and provide *β*-cell rest, a strategy that may help reduce the risk of persistent dysglycemia. Once beyond this early phase, a formal PTDM diagnosis reflects established disease, and insulin use at this stage serves as long-term glycemic management rather than prophylaxis. Our analysis integrates evidence from both early and late insulin use, underscoring the importance of tailoring therapy to the patient’s post-transplant timeline and clinical status. Nevertheless, insulin therapy requires close monitoring to minimize hypoglycemia risk and manage the burden of injections. SGLT2i represent another strong therapeutic option, combining effective glucose lowering with substantial cardiovascular and renal protective effects. Their ability to reduce major adverse cardiovascular and kidney events is particularly relevant for kidney transplant recipients, who are at elevated risk for these complications. DPP-4 inhibitors, although less potent in glycemic control, offer an excellent safety and tolerability profile, making them suitable for patients who are intolerant to other agents or who require combination therapy. Taken together, current evidence supports prioritizing insulin—particularly in the early post-transplant setting—and SGLT2i in the longer-term management of PTDM. When used appropriately, these agents have the potential to improve both patient survival and graft longevity.

This study has several limitations that warrant consideration. First, there was notable clinical and methodological heterogeneity among the included trials, including differences in patient populations, baseline characteristics, and immunosuppressive regimens, all of which may have influenced treatment responses. Second, several studies had relatively small sample sizes and markedly varied follow-up durations, which limits the ability to draw firm conclusions about long-term efficacy and safety. As with any network meta-analysis, reliance on indirect comparisons introduces an inherent risk of bias, as variations in study design, patient selection, and unmeasured confounders may affect both the precision and validity of the pooled estimates. Although our assessment suggested a low risk of publication bias, it cannot be entirely excluded. A key limitation is the scarcity of direct head-to-head trial evidence for certain drug classes in the PTDM setting—most notably SGLT2i, for which only one small RCT was available. While our findings for SGLT2i are consistent with robust evidence from non-transplant type 2 diabetes populations, the limited transplant-specific data inevitably constrains the strength of our conclusions regarding their safety and efficacy in PTDM. Future research should address these gaps with adequately powered, multicenter, randomized trials focused specifically on kidney transplant recipients.

Looking ahead, there is a critical need for large-scale, multicenter, head-to-head randomized controlled trials directly comparing antidiabetic agents in the PTDM population to validate and expand upon these findings. Future research should emphasize long-term follow-up to evaluate sustained glycemic control, cardiovascular and renal outcomes, and safety profiles. Moreover, assessing patient-centered outcomes, including quality of life and treatment adherence, will be essential to inform holistic and effective management strategies tailored to this unique patient group.

## Conclusion

This network meta-analysis highlights insulin and SGLT2i as the most effective and safe treatment options for managing PTDM. Their use may improve both glycemic control and long-term cardiovascular and renal outcomes. Further well-designed trials are needed to confirm these findings and guide optimal clinical practice.

## Data Availability

The original contributions presented in the study are included in the article/Supplementary material, further inquiries can be directed to the corresponding author.
