# Supplementary material for: Comparative efficacy and safety of antidiabetic agents for post-transplant diabetes mellitus: a network meta-analysis
Source: Front Med (Lausanne). 2025 Sep 9;12:1653147. doi: 10.3389/fmed.2025.1653147 (PMC12454430; doi:10.3389/fmed.2025.1653147)
Supplement: Supplementary file 1 [file Data_Sheet_1.docx]

**Supplemental Online Content**

**Comparative Efficacy and Safety of Blood Flow Restriction Training for Knee Osteoarthritis: A network Meta-Analysis of Randomized Controlled Trials**

1. The risk of bias summary for studies included in the meta-analysis.
2. Rankogram.
3. Funnel plot.
4. Contribution plot.
5. **The risk of bias summary for studies included in the meta-analysis.**

**a.**

**
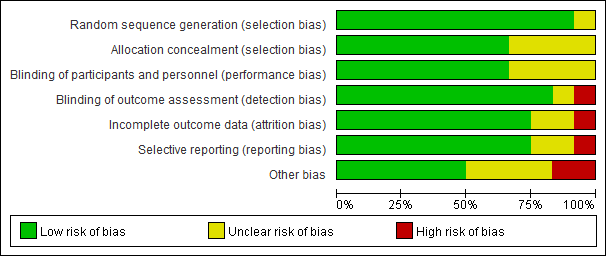
**

Risk of bias graph: review authors' judgements about each risk of bias item presented as percentages across all included studies.

b.


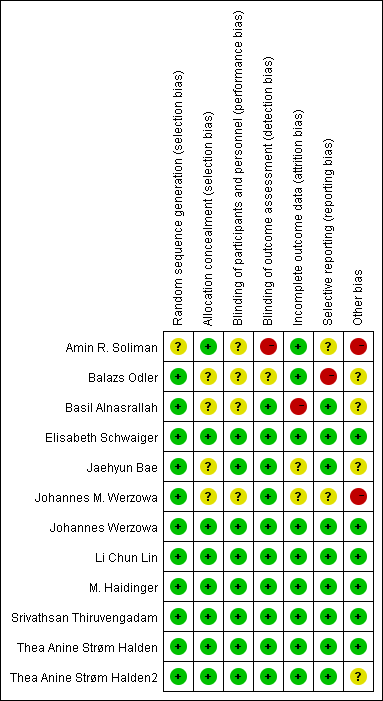


Risk of bias summary: review authors' judgements about each risk of bias item for each included study.

**2. Rankogram.**

**a.**

**b.**

**c.**

**d.**

**(a)HA1c (%); (b) FPG; (c) SBP ; (d) MACE and MAKE ;**

**3. Funnel plot**

**a.**

**b.**

**c.**

**d.**

**(a) HA1c (%); (b) FPG; (c) SBP ; (d) MACE and MAKE ;**

**4. Contribution plot.**

**a.**

**b.**

**c.**

**d.**

**(a) HA1c (%); (b) FPG; (c) SBP ; (d) MACE and MAKE ;**
